# Calcineurin inhibitors cyclosporin A and tacrolimus protect against podocyte injury induced by puromycin aminonucleoside in rodent models

**DOI:** 10.1038/srep32087

**Published:** 2016-09-01

**Authors:** Xiujin Shen, Hong Jiang, Meike Ying, Zhoutao Xie, Xiayu Li, Haibing Wang, Jie Zhao, Chuan Lin, Yucheng Wang, Shi Feng, Jia Shen, Chunhua Weng, Weiqiang Lin, Huiping Wang, Qin Zhou, Yan Bi, Meng Li, Lingyan Wang, Tongyu Zhu, Xiaoru Huang, Hui-Yao Lan, Jing Zhou, Jianghua Chen

**Affiliations:** 1 Kidney Disease Center, First Affiliated Hospital, Zhejiang University, School of Medicine; Key Laboratory of Nephropathy, Zhejiang Province, Hangzhou, Zhejiang, China; 2 National Clinical Research Base of Traditional Chinese Medicine, Zhejiang Hospital of Traditional Chinese Medicine, Zhejiang Chinese Medical University, Hangzhou, China; 3 Biomedical Research Center, Zhongshan Hospital, Fudan University, Shanghai, China; 4 Department of Urology, Zhongshan Hospital, Fudan University, Shanghai, China; 5 Li Ka Shing Institute of Health Sciences, and Department of Medicine and Therapeutics, The Chinese University of Hong Kong, Hong Kong, China; 6 Harvard Center for Polycystic Kidney Disease Research and Renal Division, Department of Medicine, Brigham and Women’s Hospital, Harvard Medical School, Boston, USA

## Abstract

Podocyte injury and the appearance of proteinuria are features of minimal-change disease (MCD). Cyclosporin A (CsA) and tacrolimus (FK506) has been reported to reduce proteinuria in patients with nephrotic syndrome, but mechanisms remain unknown. We, therefore, investigated the protective mechanisms of CsA and FK506 on proteinuria in a rat model of MCD induced by puromycin aminonucleoside (PAN) and *in vitro* cultured mouse podocytes. Our results showed that CsA and FK506 treatment decreased proteinuria via a mechanism associated to a reduction in the foot-process fusion and desmin, and a recovery of synaptopodin and podocin. In PAN-treated mouse podocytes, pre-incubation with CsA and FK506 restored the distribution of the actin cytoskeleton, increased the expression of synaptopodin and podocin, improved podocyte viability, and reduced the migrating activities of podocytes. Treatment with CsA and FK506 also inhibited PAN-induced podocytes apoptosis, which was associated with the induction of Bcl-xL and inhibition of Bax, cleaved caspase 3, and cleaved PARP expression. Further studies revealed that CsA and FK506 inhibited PAN-induced p38 and JNK signaling, thereby protecting podocytes from PAN-induced injury. In conclusion, CsA and FK506 inhibit proteinuria by protecting against PAN-induced podocyte injury, which may be *associated with inhibition of the* MAPK signaling pathway.

Minimal-change disease (MCD) is one of the primary glomerular diseases, and proteinuria is the main clinical manifestation. The development of proteinuria is due to podocyte injury that damages the integrity of the glomerular filtration barrier. An early event in podocyte injury is the decreased expression of podocyte cytoskeletal proteins such as synaptopodin, nephrin, and podocin, which results in disorganization of the cytoskeleton and the fusion of foot processes and leads to the development of proteinuria and subsequent kidney damage^1^,^2^.

Corticosteroid is a mainstay of treatment for MCD. However, the prolonged use of steroids also causes a severe side-effect clinically^3^. Recently, calcineurin (CaN) inhibitors such as cyclosporin A (CsA) and tacrolimus (FK506) were found to effectively reduce the development of proteinuria in patients with nephrotic syndrome^4^,^5^,^6^, although both drugs are recognized as immunosuppressants and have been widely used to prevent the rejection of kidney transplants. Recent studies also showed that CsA and FK506 are beneficial in treating patients with steroid-resistant nephrotic syndrome^7^,^8^,^9^. It has been reported that CsA is beneficial in nephritic syndrome caused by the Wilm’s tumor-1 (WT-1) mutation in podocytes^5^. Treatment with FK506 decreases the proteinuria in patients with IgA nephropathy and membranous nephropathy^4^,^6^. Our previous studies also showed that FK506 has a higher remission rate than cyclophosphamide in treating nephrotic syndrome with adult hormone resistance^10^,^11^. However, the molecular mechanisms by which CsA and FK506 reduce proteinuria are still not fully understood. Faul *et al.*^12^ first showed that CsA directly protects the podocyte cytoskeleton by inhibiting the expression of CaN in podocytes to decrease the cathepsin L-dependent cleavage of synaptopodin, suggesting that podocytes might be the target of CaN inhibitors in reducing proteinuria.

Apoptosis is a major pathological feature of podocytes injury and can be induced by puromycin aminonucleoside (PAN), angiotensin II, and endothelin-1^13^,^14^. Once podocytes injury occurs, proteinuria develops, resulting in the progression to renal failure^15^. It has been reported that podocyte apoptosis is associated with the activation of CaN. Treatment with FK506 and CsA can block the apoptosis induced by angiotensin II, endothelin-1 and adriamycin^13^,^14^. MAPK (mitogen-activated protein kinase) signaling pathway plays an important role in podocyte apoptosis^16^,^17^,^18^. It has also been reported that treatment with FK506 inhibits phosphorylation of the JNK and p38 pathways in human rheumatoid fibroblast-like synoviocytes and mouse macrophages in response to IL-1β and LPS treatment^19^,^20^. However, it remains unknown if the renoprotective effect of FK506 and CsA on proteinuria is associated with inhibition of podocytes apoptosis via the MAPK signaling pathway. Thus, in the present study, we tested the hypothesis that FK506 and CsA may inhibit proteinuria by protecting against podocytes apoptosis via a mechanism associated with MAPK pathway in a PAN-induced nephrotic rat model *in vitro*.

## Results

### CsA and FK506 alleviate PAN-induced nephrotic syndrome in rats by protecting against podocyte damage

**Figure 1:**
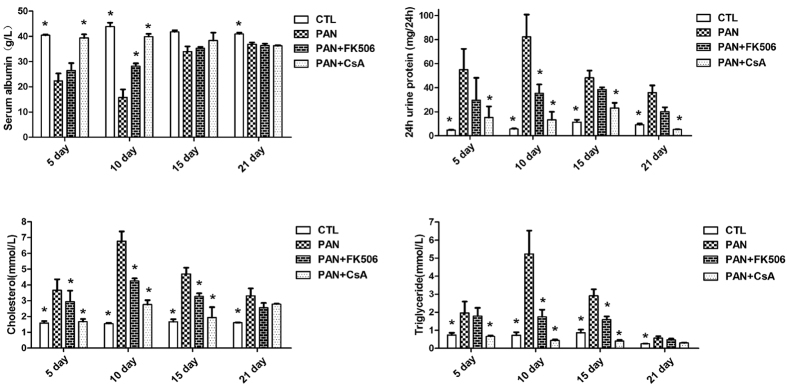
CsA and FK506 ameliorate proteinuria, and serum albumin, triglyceride, and cholesterol abnormalities in SD rats. CsA and FK506 reduced the 24-h urinary protein, decreased the triglyceride and cholesterol levels, and restored the serum albumin level in PAN-treated rats. **CTL**, normal rats; **PAN**, PAN-treated rats; **PAN + FK506**, intragastric administration of FK506 starting at the same time as PAN injection; **PAN + CsA**, intraperitoneal injection of CsA starting at the same time as PAN injection (n = 5 per group; *P < 0.05 *vs* PAN group).

As shown in Fig. 1, severe proteinuria, hyperlipidemia, hypercholesterolemia, and hypoalbuminemia were developed in PAN-treated rats, which were significantly inhibited by treatment with either CsA or FK506.

**Figure 2:**
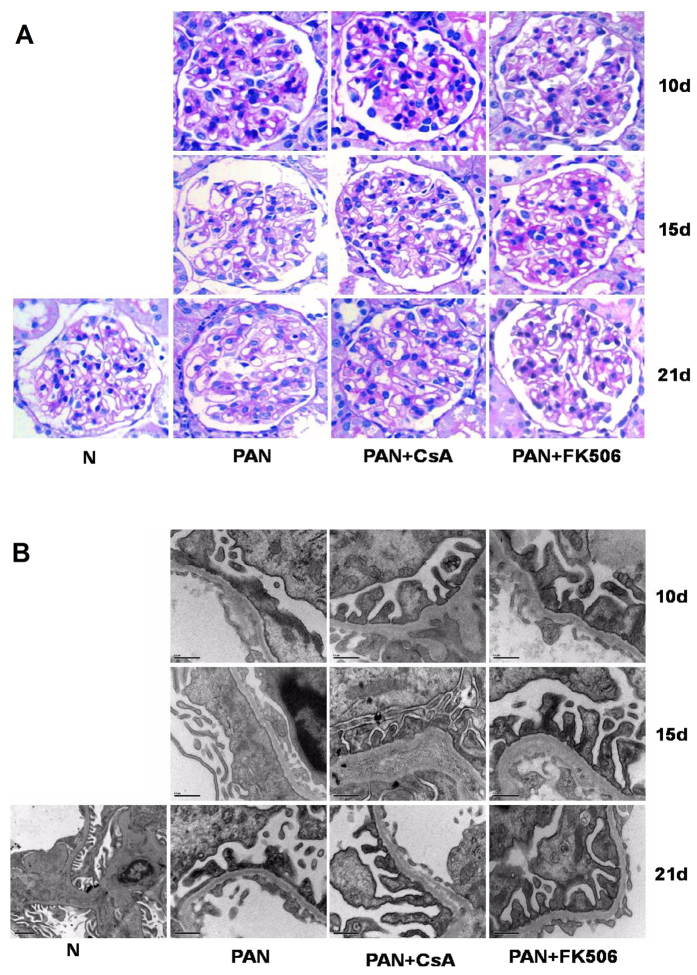
Glomerular morphology and foot processes in normal, PAN, PAN + FK506, and PAN + CsA treated SD rats. (**A**) PAS staining showed no difference in glomerular morphology between groups at indicated stages after CsA and FK506 treatment in PAN injured SD rats. Original magnification, X400. (**B**) Transmission electron microscopy showed extensive foot-process effacement at 10 and 15 days after PAN injection. CsA and FK506 treatment significantly decreased foot-process width compared with PAN-only rats. Scale bar, 0.5 μm (5 μm in normal rats). Images shown are representative from 5 rats for each treatment group.

Histologically, Periodic acid-Schiff (PAS) staining detected no significant changes between treated and untreated animals (Fig. 2A). However, electron microscopy found that the characteristics of podocyte injury such as foot-process fusion, and the absence of slit diaphragms were observed in glomeruli of PAN-treated rats, becoming apparent over days 10–15 with partially recovered after PAN treatment. In contrast, treatment with CsA or FK506 obviously reversed the foot-process effacement (Fig. 2B). Quantitatively, CsA and FK506 treatment significantly reduced the mean foot-process width over the entire disease course (Table 1).

**Figure 3:**
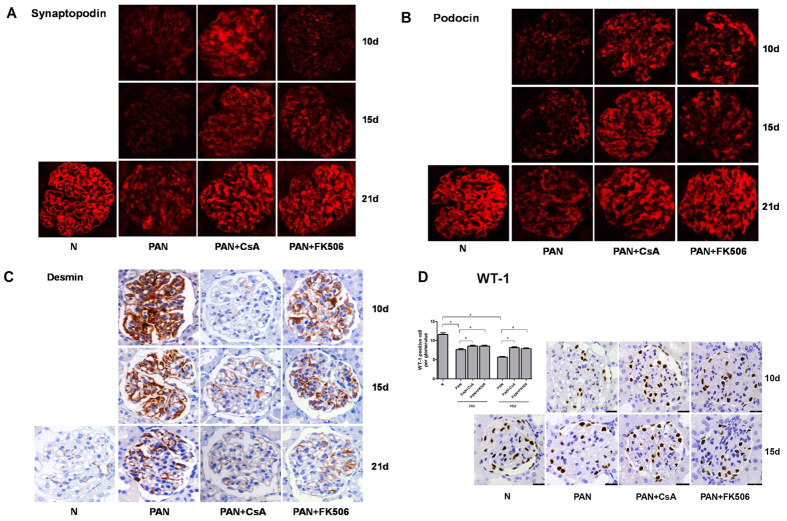
Expression of synaptopodin, podocin, desmin and WT-1 in kidney glomerulus from SD rats subjected to various treatments. Immunofluorescent and immunohistochemical staining for synaptopodin (**A**), podocin (**B**), desmin (**C**) and WT-1(D) showed that CsA treatments rescued the expression of synaptopodin, podocin and WT-1 in PAN-treated SD rats and inhibited PAN induced desmin expression. Original magnification, X400. Images shown are representative from 5 rats for each treatment group. WT-1 positive podocytes were calculated from more than 50 glomeruli (*P < 0.05).

We next examined expression levels of synaptopodin and podocin, two common biomarkers for podocyte, and found that treatment with CsA and FK506 gradually restored the normal distribution and expression of synaptopodin and podocin when compared to the PAN-treated rats, becoming normal at 21 days after treatment (Fig. 3A,B). In addition, we found that treatment with CsA and FK506 also promoted the podocyte repair process by significantly inhibiting expression of desmin in PAN-treated rats (Fig. 3C). WT-1 is a specific marker for podocytes. In order to detect the podocyte number, we calculated WT-1 positive cells in glomeruli (Fig. 3D). Our results showed treatment with CsA and FK506 obviously recovered the number of WT-1 positive cells at 10 and 15 days, implying that CsA and FK506 might play an important role in maintaining podocyte viability or inhibiting podocyte apoptosis.

### CsA and FK506 protect against PAN-induced podocyte injury *in vitro*

**Figure 4:**
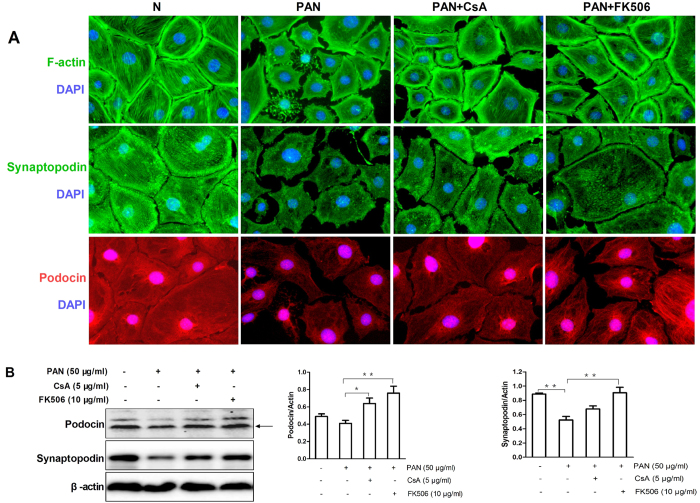
CsA and FK506 pretreatment protect against PAN-induced injury in cultured mouse podocytes *in vitro*. (**A**) Immunofluorescence of F-actin, synaptopodin and podocin in PAN treated mouse podocytes after CsA and FK506 treatment. Original magnification, X400. (**B**) Western blot analyses of podocin and synaptopodin in PAN injured mousepodocytes after CsA and FK506 treatment for 24 h (podocin: N 0.49 ± 0.05, PAN 0.4 ± 0.06, PAN + CsA 0.6 ± 0.1, PAN + FK506 0.76 ± 0.14; synaptopodin: N 0.89 ± 0.03, PAN 0.52 ± 0.1, PAN + CsA 0.67 ± 0.09, PAN + FK506 0.9 ± 0.15; n = 3; *P < 0.05, **P < 0.01). All experiments were performed for 3 times, and the results were shown as mean ± SD.

**Figure 5:**
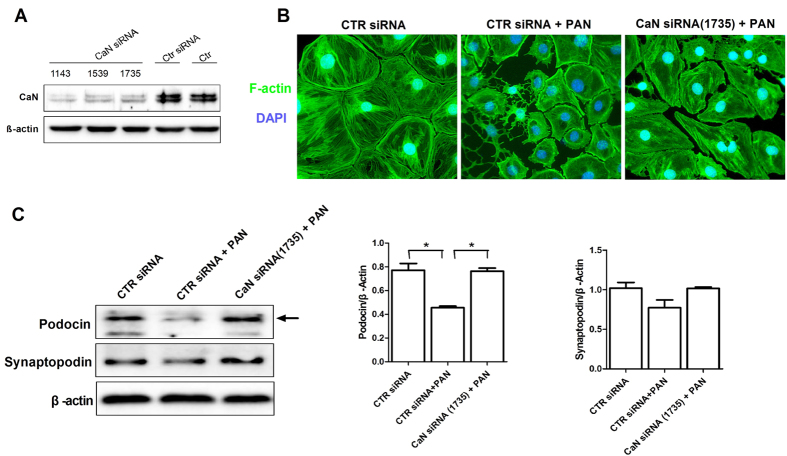
CaN inhibition protect against PAN-induced injury in cultured mouse podocytes *in vitro*. **(A)** Western blot analysis of CaN. (**B)** Immunofluorescence of F-actin in PAN treated mouse podocytes after CaN siRNA treatment. Original magnification, X400. **(C)** Western blot analyses of podocin and synaptopodin in PAN injured mouse podocytes after CaN siRNA treatment for 24 h (*P < 0.05). All experiments were performed for 3 times, and the results were shown as mean ± SD.

As reorganization of the cytoskeleton is a characteristic of podocyte injury, we assessed the morphological changes of podocyte cytoskeleton, together with the expression of podocin and synaptopodin, in mouse podocytes in response to the PAN. Our results showed that PAN caused podocyte retraction and disorganization of the actin stress fibers, which were associated with inhibition of synaptopodin and podocin expression (Fig. S2A,B). In contrast, pretreatment of podocytes with either CsA or FK506 reversed PAN-induced disorganization of the cytoskeleton and downregultion of podocin and synaptopodin (Fig. 4A,B). However, treatment with CsA or FK506 alone had no influence on the cytoskeleton or the expression of synaptopodin and podocin (Fig. S1B). CaN is the common target of both CsA and FK506. Our results showed that CaN inhibition by siRNA (Fig. 5A) restored the expression of F-actin, synaptopodin and podocin (Fig. 5A), indicating that CaN plays an important role in the protective effect of CsA and FK506.

**Figure 6:**
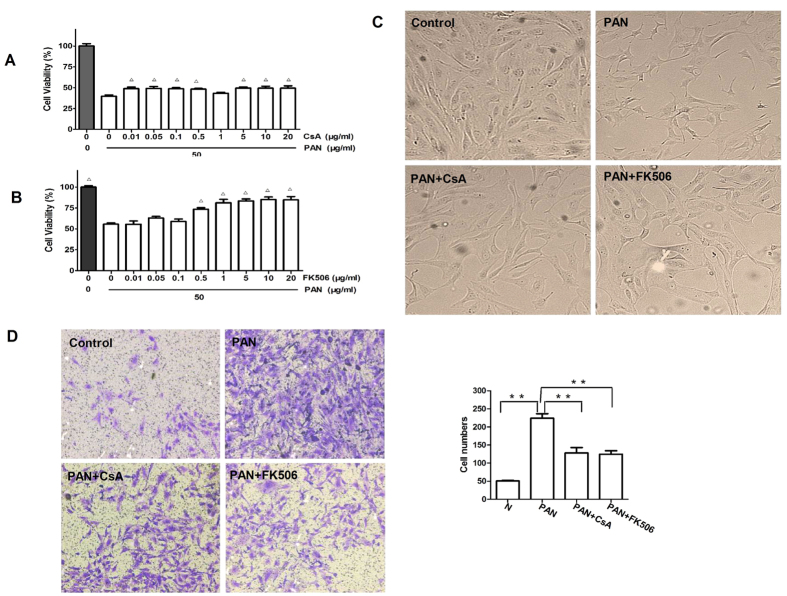
CsA and FK506 pretreatment enhance viability and decrease motility in PAN-treated podocytes *in vitro*. MTT assays of podocyte viability 24 h after CsA (**A**) or FK506 (**B**) treatment at indicated concentrations. Viability in the control group (0.1% DMSO) was set at 100% (^Δ^p < 0.05 *vs* PAN group). (**C**) Light microscopy of podocyte morphology 48 h after CsA and FK506 treatment. Original magnification, X200. (**D**) Transwell migration assays of podocyte motility 24 h after CsA and FK506 treatment (**P < 0.01). Original magnification, X100. All experiments were performed at least 3 times, and the results were shown as mean ± SD.

In order to find out the effects of injured podocyes on podocyte function, we examined the viability of cultured mouse podocytes using MTT assays. Results showed that addition of PAN clearly reduced the podocyte viability, which was prevented by pre-incubation of podocytes with CsA (Fig. 6A) or FK506 (Fig. 6B). Interestingly, treatment of podocytes with CsA or FK506 alone for 24 h slightly increased the proliferative activities (Fig. S1A). In contrast, PAN treatment for 48 h resulted in a reduction in the podocyte number, which was again prevented by CsA or FK506 pretreatment (Fig. 6C). Subsequently, we examined the motility of cultured mouse podocytes by transwell migration assays and found that PAN treatment for 24 h significantly increased the number of migrating podocytes, which was blunted when cells were pretreated CsA or FK506 (Fig. 6D). There was no difference between CsA and FK506 in promoting podocyte motility.

### CsA and FK506 reduce PAN-induced podocyte apoptosis *in vitro*

**Figure 7:**
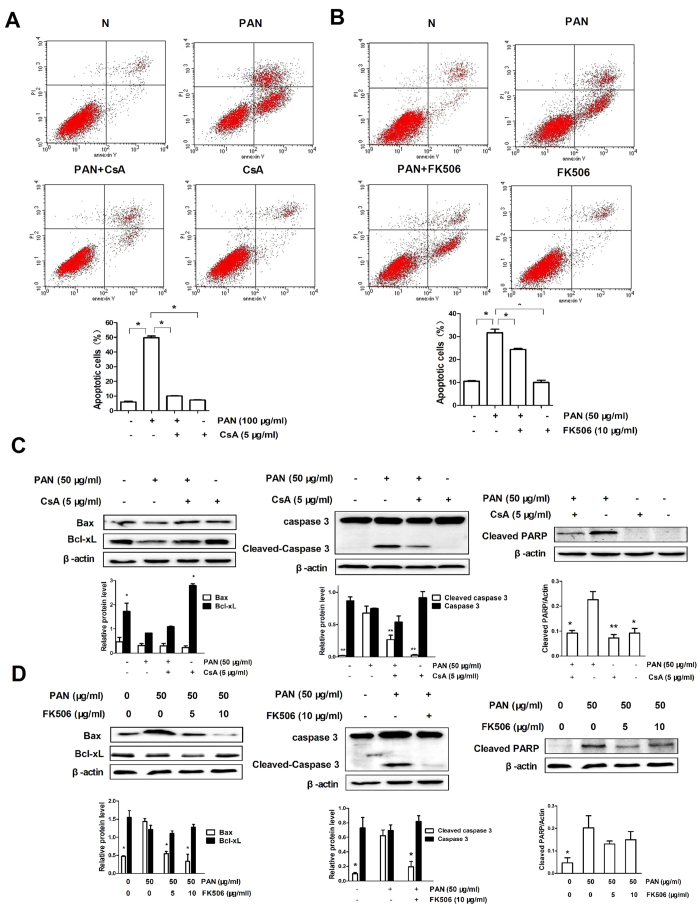
CsA and FK506 inhibit PAN-induced podocyte apoptosis in cultured mouse podocytes *in vitro*. (**A,B**) Flow cytometric analysis of the effects of CsA and FK506 pretreatment on PAN-induced apoptosis (*P < 0.05). All experiments were performed for 3 times, and the results were shown as mean ± SD. (**C**,**D**) Expression of Bax, Bcl-XL, cleaved caspase 3, and cleaved PARP after 5 μg/ml CsA (**C**) and 10 μg/ml FK506 (**D**) treatment for 24 h in PAN-treated podocytes (* < 0.05, **P < 0.01 *vs* PAN group). All experiments were performed for 3 times, and the results were shown as mean ± SD.

**Figure 8:**
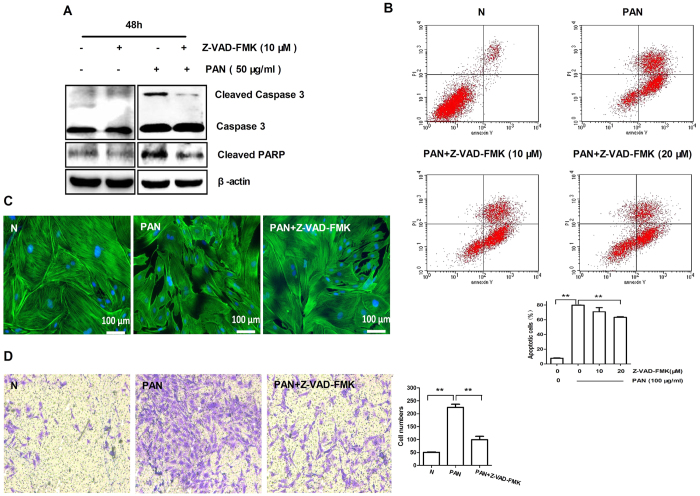
Z-VAD-FMK protects podocytes against PAN-induced apoptosis in cultured mouse podocytes *in vitro*. (**A**) Western blot analysis of cleaved caspase 3 and cleaved PARP. (**B**) Flow cytometric analysis of apoptosis after Z-VAD-FMK treatment (**P < 0.01). (**C**) Immunofluorescence of F-actin in PAN treated mouse podocytes after Z-VAD-FMK treatment (PAN: 50 μg/ml; Z-VAD-FMK: 10 μM). Scale bar, 100 μm; (**D**) Transwell migration assays of podocyte motility after Z-VAD-FMK treatment (**P < 0.01; original magnification, X100). All experiments were performed for 3 times, and the results were shown as mean ± SD.

It is believed that persistent podocyte injury leads to apoptosis. We next examine whether CsA and FK506 protect against podocytes injury by inhibiting their apoptosis. Flow cytometric analysis showed that addition of PAN markedly enhanced podocyte apoptosis in a dose-dependent manner, which was prevented by pretreatment with either CsA or FK506 (Fig. 7A,B). In addition, we also found that addition of PAN also induced podocyte apoptosis by inhibiting expression of Bcl-2 and Bcl-XL, but increasing Bax, cleaved caspase-3, and cleaved PARP in a dose-dependent manner (Fig. S3). In contrast, pretreatment of podocytes with CsA or FK506 inhibited PAN-induced Bax, cleaved PARP, and cleaved caspase-3, but significantly increased Bcl-XL expression (Fig. 7C,D). To assess the role of the caspase family in apoptosis, we pretreated podocytes with the general caspase inhibitor Z-VAD-FMK. Results showed that treatment with Z-VAD-FMK significantly reduced PAN-induced apoptosis pathway including the expression of cleaved caspase-3 and cleaved PARP (Fig. 8A,B), disorganization of the cytoskeleton, (Fig. 8C), and podocytes migration (Fig. 8D). These results demonstrated that CsA and FK506 suppress podocyte apoptosis by inhibiting mitochondria-dependent pathways.

### CsA and FK506 reduces PAN-induced podocyte mitochondrial dysfunction *in vitro*

**Figure 9:**
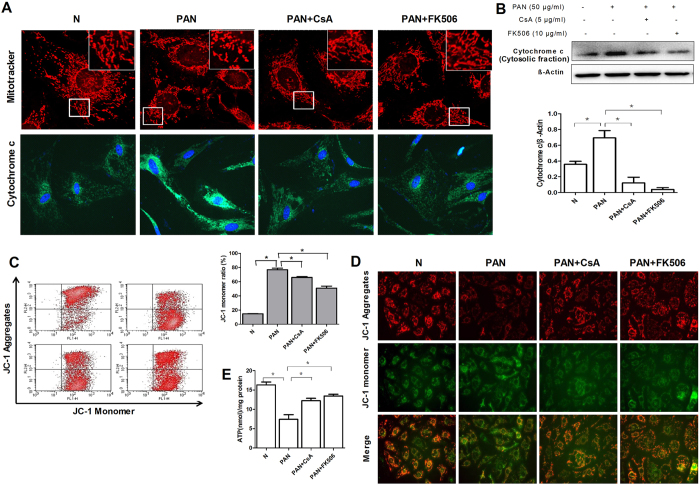
CsA and FK506 inhibit PAN-induced disorders of mitochondrial function in cultured mouse podocytes *in vitro*. **(A**) Fluorescent staining of podocyte mitochondria with Mito Tracker Red CMXRos after CsA and FK506 treatment for 24 h in PAN-treated podocytes, the expression of cytochrome c was also detected. **(B)** Quantification also indicated increased expression of cytochrome c (cytosolic fraction) was inhibited by CsA and FK506. **(C)** Flow cytometric analysis and fluorescent staining **(D)** of the effects of CsA and FK506 pretreatment on PAN-induced MMP loss in podocytes (*P < 0.05). **(E)** Flow cytometric analysis of the effects of CsA and FK506 pretreatment on PAN-induced ATP reduction in podocytes (*P < 0.05). All experiments were performed for 3 times, and the results were shown as mean ± SD.

Previous reports have shown that PAN induced mitochondrial dysfunction was related to podocyte apoptosis^21^,^22^,^23^,^24^. Our study identified that the protective effect of CsA and FK506 on podocyte apoptosis was associated with the inhibition on the release of cytochrome c (cytosolic fraction) induced by PAN (Fig. 9A,B). Accumulation of damaged mitochondria may lead to apoptosis. Mitochondria staining indicated that the normal mitochondria in podocytes was thin and elongated, mitochondria became short and fragmented in PAN induced podocytes, which was reversed by CsA or FK506 preincubation (Fig. 9A). JC-1 staining also showed that CsA and FK506 pretreatment inhibited the decrease in mitochondrial membrane potential (MMP) in PAN-induced podocytes (Fig. 9C,D). Further studies showed that the reduction of ATP level in podocytes was increased after CsA and FK506 pretreatment (Fig. 9E).

**Figure 10:**
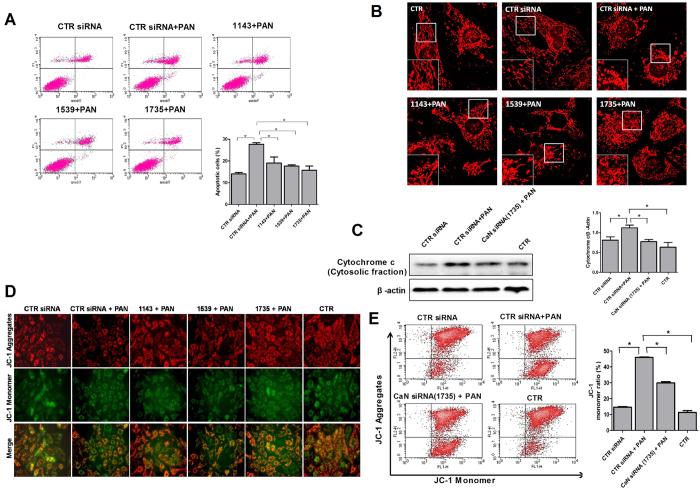
Calcineurin inhibition protects podocytes against PAN-induced mitochondria dependent apoptosis. **(A)** Flow cytometric analysis of podocyte apoptosis with or without CaN siRNA after PAN treatment (*P < 0.05). **(B)** Podocyte mitochondria was visualized using MitoTracker Red CMXRos. Images were taken with confocal microscope. **(C)** Western blot analysis of cytochrome c (cytosolic fraction) with or without CaN siRNA after PAN treatment. **(D)** Fluorescent staining and flow cytometric analysis **(E)** of the effects of CaN siRNA on PAN-induced MMP in podocytes (*P < 0.05). All experiments were performed for 3 times, and the results were shown as mean ± SD.

In order to better understand the effect of calcineurin inhibition on PAN-induced mitochondrial dysfunction in podocytes, CaN siRNA was used. As shown in Fig. 10A,C, CaN siRNA treated podocytes indicated a significant reduction of apoptosis and cytochrome c expression (cytosolic fraction) compared to control siRNA(CTR siRNA). Mitochondria morphology and MMP were also reversed by CaN siRNA in PAN treated podocytes (Fig. 10B,D,E). According to these findings, it is likely that calcineurin is involved in mitochondria dependent podocyte apoptosis, and the anti-apoptotic effects of CsA and FK506 are partly related to calcineurin.

### MAPK signaling pathway is involved in the protective effect of CsA and FK506 on podocytes apoptosis

**Figure 11:**
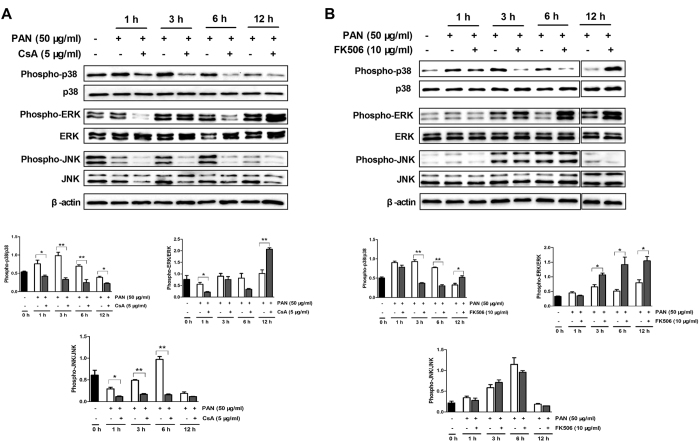
MAPK signaling pathway is involved in the protective effects of CsA and FK506 on cultured mouse podocytes *in vitro*. (**A**) CsA pretreatment (5 μg/ml) inhibited phospho-p38 and phospho-JNK activation while activating phospho-ERK in PAN-treated podocytes (*P < 0.05, **P < 0.01). (**B**) FK506 pretreatment (10 μg/ml) inhibited phospho-p38 and activated phospho-ERK in PAN-treated podocytes, but had no effect on the activation of phospho-JNK (*P < 0.05, **P < 0.01). All experiments were performed for 3 times, and the results were shown as mean ± SD.

In order to explore the molecular mechanisms underlying the protective effect of CsA and FK506 on podocyte apoptosis, we examined phosphorylation levels of p38, ERK, and JNK in response to PAN. Results indicated that addition of CsA or FK506 significantly increased the phosphorylation levels of ERK without affecting p38 and JNK activation. Moreover, PAN showed a rapid and transient increase in p38 and JNK phosphorylation (Fig. S4), which was inhibited by pretreatment with CsA from 1 h to 6 h (Fig. 11A). Interestingly, FK506 pretreatment effectively suppressed PAN-induced p38 phosphorylation and increased PAN-induced ERK phosphorylation without altering PAN-induced phosphorylation of JNK (Fig. 11B).

**Figure 12:**
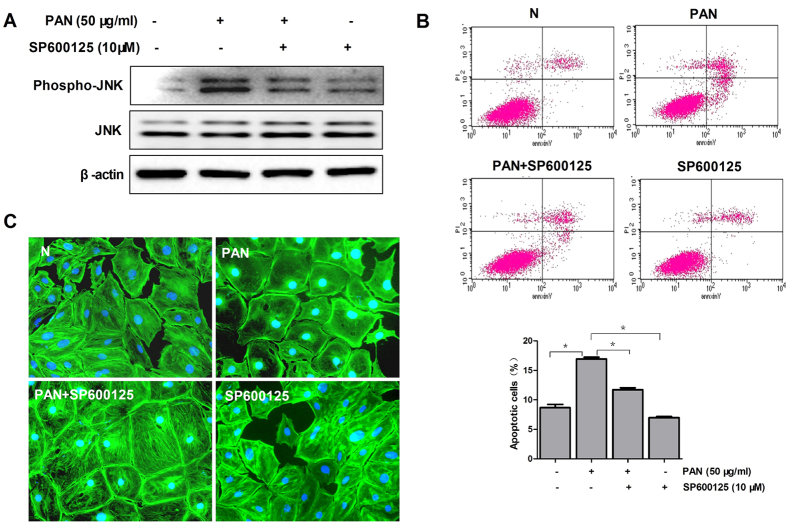
Role of the JNK signaling pathway in podocyte protection. **(A)** Western blot analysis of Phospho-JNK and JNK before and after SP600125 (JNK inhibitor) incubation in PAN treated podocytes (6 h after incubation). **(B)** Flow cytometric analysis of podocyte apoptosis with or without SP600125 after PAN treatment (*P < 0.05). The experiments were performed for 3 times, and the results were shown as mean ± SD. (**C**) Immunofluorescence of F-actin in PAN treated mouse podocytes after SP600125 incubation. Original magnification, X400.

JNK is known to promote the mitochondrial death signaling pathway. We depleted podocytes of JNK with SP600125 (Fig. 12A) and treated them with PAN. Our results showed that JNK inhibition significantly reduced PAN induced podocyte apoptosis (Fig. 12B) and cytoskeleton disarrangement (Fig. 12C) at 6 h after incubation. Therefore, our observations demonstrated that JNK is involved in PAN induced apoptosis, and the pro survival effect of calcineurin inhibitors CsA and FK506 may be a result by interfering with JNK activity.

## Discussion

Proteinuria is the common feature of nephrotic syndrome (including MCD) and is induced by the podocyte injury^2^,^25^. Therefore, podocyte protection is effective in inhibiting proteinuria. In this study, we reported that CaN inhibitors (CsA and FK506) inhibited proteinuria in PAN-induced nephrotic syndrome in rats by protecting against podocyte injury via mechanisms associated with reducing the foot-process effacement, reversing the disorganization of cytoskeleton and the loss of podocyte phenotype, and inhibiting the podocyte apoptosis in models of PAN-induced injury.

Recent studies indicated that non-immunological factor of calcineurin inhibitors may directly affect proteinuria in nephrotic syndrome by regulating the stability of podocyte cytoskeleton^6^,^12^. In the present study, we found CaN inhibitors (CsA and FK506) reduced the 24-h urinary protein and ameliorated the serum albumin, triglyceride, and cholesterol abnormalities in PAN-treated SD rats, as well as reducing the foot-process effacement and recovering the expression of podocyte cytoskeleton markers synaptopodin and podocin, implying that CsA and FK506 reduced proteinuria by protecting podocytes in PAN induced MCD model. Furthermore, in agreement with previous findings^6^,^26^,^27^,^28^, we also found that CsA and FK506 directly protect podocyte cytoskeleton and restore the expression of synaptopodin and podocin in cultured mouse podocytes. Moreover, podocyte functional assay also showed increased cell viability and decreased cell motility in CsA and FK506 preincubated podocytes. The protective effects of CaN inhibitors (CsA and FK506) were also observed in other podocyte injury models such as adriamycin induced MCD^29^, membranous nephropathy^4^, Lupus Nephritis^27^ and IgA nephropathy^6^.

FK506 plus low-dose steroids has been reported to have similar efficacy, lower risk of relapses, and fewer cosmetic side-effects in patients with steroid-resistant nephrotic syndrome than CsA combined with steroids^30^. We also compared the effect of FK506 with CsA and our results indicated that FK506 protect podocytes similarly to CsA, and the effects of FK506 in alleviating proteinuria, and normalizing serum albumin, triglyceride, and cholesterol was not better than CsA *in vivo*. Similarly, Wang L. *et al.*^31^ also found that FK506 had non-significant effects on the development of focal segmental glomerulosclerosis.

A consequence of podocyte injury is apoptosis, leading to podocyte depletion and subsequent glomerulosclerosis. Inhibition of podocyte apoptosis is usually speculated to promote podocyte survival. PAN treatment is a well-established model of podocyte apoptosis, which is related to mitochondria dependent pathway. Our *in vivo* results showed that PAN induced podocyte loss was reversed by CsA and FK506 preincubation (Fig. 3D), implying that CsA and FK506 might play an important role in maintaining podocyte viability by inhibiting podocyte apoptosis. To determine whether CsA and FK506 alleviate podocyte injury by reducing mitochondria dependent apoptosis, we preincubated PAN-treated podocytes with CsA or FK506 *in vitro*. Our results, consistent with previous findings^14^,^27^,^32^, reported the anti-apoptotic effects of FK506 on podocytes. However, the effects of CsA on podocyte apoptosis are controversial. Fornoni *et al.*^33^ firstly demonstrated that CsA induced podocyte apoptosis in a podocyte cell line. However, our results indicated that CsA showed an anti-apoptotic effect in injured podocytes, other researchers also failed to reproduce the proapoptotic effect of CsA^12^,^13^,^34^ the origin of the cell lines used in experiments might be the reason. Our findings showed for the first time that both CsA and FK506 reversed the elevation of Bax expression and reduced the Bcl-XL expression in PAN-treated podocytes. Meanwhile, our results showed that both CsA and FK506 decreased caspase 3 activation and subsequent PARP cleavage in PAN-treated podocytes, as well as reversing the PAN-induced apoptosis and migration by Z-VAD-FMK. Furthermore, we also found that CsA and FK506 alleviated PAN-induced podocyte mitochondrial dysfunction. Calcineurin inhibition with CaN siRNA also proved that calcineurin might be one of the targets of mitochondria dependent apoptosis in podocytes, suggesting that CaN inhibitors might play a crucial role in preventing mitochondria-dependent podocyte apoptosis.

The *in vitro* results showed that the podocyte cytoskeletal disorder occurs first when incubated with PAN, podocytes shrink emerged subsequently (arrow shows the shrunk podocyte), at last, podocytes apoptosis began to appear (Figure S2A). The *in vivo* results also showed the podocyte loss after PAN incubation (the number of WT-1 positive cell in the glomerulus), which was recovered by calcineurin inhibitors, implying that the calcineurin inhibitors could protect podocyte both by cytoskeletal reorganization and partially by inhibition of podocyte apoptosis.

It has been demonstrated that the anti-proteinuria effect of CaN inhibitors are associated with the inhibition of the NFAT signaling pathway in T cells as well as podocytes^28^,^31^,^35^,^36^,^37^. In addition to the calcineurin-NFAT pathway, previous studies showed evidences that MAPK signaling, especially p38 and JNK pathways also play vital role in the action for CsA and FK506^38^,^39^,^40^,^41^. The MAPK signaling pathway has been shown to mediate podocyte injury, apoptosis and proteinuria^42^,^43^,^44^. Moreover, they were also associated with disruption of the actin cytoskeleton in podocytes^17^,^44^,^45^,^46^. Thus, the relative extents of MAPK activation were speculated to determine podocyte fate.

It has been demonstrated that the activation of p38^43^,^44^,^45^ and JNK^47^,^48^ participate in podocyte injury. Our results also revealed that pretreatment with CsA and FK506 reduced the PAN-induced phosphorylation of p38 pathways. Unexpectedly, we found FK506, rather than CsA, increased the activation of p38 in PAN induced podocytes at 12 h, other signaling pathway might play a vital role in the later stage of FK506 treatment, other than CsA preincubation, which made the re-phosphorylation of p38 pathways. Furthermore, our findings showed that CsA, contrast with FK506, indicated a negative effect with the PAN-induced JNK activation, which was consistent with previous studies of glomerular mesangial cells^49^ and vascular smooth muscle cells^50^. JNK is known to promote the mitochondrial death signaling pathway. It has been shown that JNK inhibitor significantly decreased podocyte apoptosis^18^,^42^,^51^, Our results also found that JNK was involved in PAN induced cytoskeleton re-arrangement and apoptosis, suggesting that the pro-survival effect of CsA may be a result by interfering with JNK activity. However, the lack of JNK inhibition by FK506 implied that FK506 might protect podocytes from PAN induced mitochondria dependent apoptosis through a pathway independent from calcineurin, which share the same target with CsA. Matsuda *et al.*^40^ also showed an inhibition of JNK signaling pathway by calcineurin inhibitors via calcineurin-independent mechanisms, indicating that MAPK signaling pathway might be an additional target of CsA and FK506 in addition to calcineurin. Further studies are needed to determine the distinct mechanisms between CsA and FK506 on their anti-apoptotic effect.

In the present study, we observed an obvious activation of ERK in PAN treated podocytes at 12 h, which was further activated by CsA or FK506 preincubation. Several studies from other groups also demonstrated that CsA and FK506 increase the activation of ERK in renal mesangial cell^38^ and proximal tubular cells^41^. ERK activation is closely relevant to podocyte injury and apoptosis^17^,^43^,^52^. It was shown that the MEK inhibitor U0126 effectively prevented podocyte apoptosis and protect podocyte cytoskeleton^17^,^18^. Furthermore, O’Connell *et al.*^53^ demonstrated that the activation of ERK accelerated the CsA induced HMC (human renal mesangial cells) toxicity, implying that the ERK activation of CsA and FK506 may accelerate podocyte injury, however, there was some controversial result showed decreased ERK phosphorylation in PAN injured podocytes^54^, further study needs to be proved for the actual effect of ERK in podocytes.

Faul *et al.*^12^ was the first to show the directly protective effect of CsA on podocyte cytoskeleton. We confirm that both CsA and FK506 protect podocyte directly. Our observation in Fig. S2A implied that podocyte apoptosis and the change of related signaling pathways are probably a consequence of the degradation/restoring of synaptopodin and disturbed actin cytoskeleton organization which made podocyte susceptible to cell death.

In conclusion, our study revealed the protective effects of the CaN inhibitors CsA and FK506 on PAN-induced proteinuria and podocyte injury *in vitro* and *in vivo*. We also found CsA and FK506 inhibited mitochondria dependent podocyte apoptosis, which might be mainly due to the different regulation of MAPK signaling pathway, and such differences might contribute to diverse clinical effects of these agents.

## Methods

### Reagents and antibodies

PAN (Cat. P7130), FK506 (Cat. F4679) (for cell culture), CsA (Cat. C3662) (for cell culture), MTT (Cat. M2128), DAPI (Cat. D9542) and anti-FITC-phalloidin antibody (Cat. P5282) were from Sigma (St Louis, MO, USA). For animal experiments, FK506 was from Fujisawa Pharmaceutical (Tokyo, Japan), CsA from Novartis Pharmaceutical (Basel, Switzerland). The FITC-Annexin V apoptosis detection kit (Cat. 556547) and JC-1 detection kit (Cat. 551302) were both from Becton Dickinson (San Diego, CA, USA). The ATP Detection kit (Cat. S0026) and crystal violet were from Beyotime Institute of Biotechnology (Shanghai, China). The Mito Tracker Red CMXRos (Cat. 9082S) was from Cell Signaling (Boston, MA, USA). The SP600125 (Cat.S1460) was from Selleck (Boston, MA, USA). Collagen Type I (Cat.354236) was from Corning (Corning, NY, USA). Z-VAD-FMK (Cat. ALX-260-020) from ENZO (New York, NY, USA), mouse recombinant IFN-γ (Cat. 34–8311) from eBioscience (San Diego, CA, USA). Antibodies for the experiment were shown at Table S1. The secondary antibody kit (GK500705) for immunohistochemistry was from Gene Co. (Shanghai, China), and the secondary antibodies for immunofluorescence were from Invitrogen (Carlsbad, CA, USA).

### Ethics statement

The experimental protocols were approved by the Ethics Committee of the First Affiliated Hospital, Zhejiang University, School of Medicine. All experiments were conducted in accordance with approved guidelines of the First Affiliated Hospital, Zhejiang University, School of Medicine.

### Animal experiments

Male Sprague-Dawley rats (Shanghai Laboratory Animal Center, Chinese Academy of Sciences, Shanghai, China) weighing 180–200 g were fed a standard diet. The rat model of MCD was induced by a single intravenous injection (i.v.) of PAN (7 mg/100 g body weight), and proteinuria was begun to detect 4 days later. The rats were randomly divided into 4 groups: (1) normal rats (n = 5); (2) PAN treatment (n = 5); (3) PAN + FK506 treatment (n = 5); and (4) PAN + CsA treatment (n = 5). In the treatment groups, FK506 (2 mg/kg/day by intragastric administration (i.g.) and CsA (20 mg/kg/day by intraperitoneal injection (i.p.) were started at the same time as PAN injection. At 5, 10, 15, and 21 days, 24-h urine was collected using metabolic cages, and serum was taken from each group for biochemical analysis. Rats were killed after anesthesia with pentobarbital sodium. Markers in renal tissue were detected by immunofluorescence and electron microscopy. Twenty-four-hour urinary protein concentrations were measured using an Aeroset biochemical analyzer (Abbott, Chicago, USA). Serum albumin, creatinine, cholesterol, and triglyceride levels were measured using a Hitachi biochemical analyzer (Hitachi 7600, Tokyo, Japan).

### Transmission electron microscopy

Renal cortex samples from each group (l mm^3^) were fixed in 2.5% glutaraldehyde, washed in PBS, fixed in 2% osmium tetroxide for 2 h, dehydrated in graded acetone and ethanol, and embedded in epoxy resin. Ultrathin sections (80–90 nm) were stained with uranyl acetate and lead citrate, then examined and photographed under Olympus transmission electron microscope (Tecnai, Tokyo, Japan).

### Measurement of foot-process width

To calculate the mean foot-process width, each image was analyzed using SimplePCI software (CompixCo., Irvine, CA, USA). The number of slit diaphragms was counted and the curved length of the peripheral capillary basement membrane was measured. Wp (average foot-process width, nm) was calculated as (π/4) × (∑GBM length / ∑number of foot processes).

### Immunofluorescence and immunohistochemistry of rat kidney

After de-paraffinization and rehydration, the 1.5-μm biopsy specimens were incubated with rabbit anti-synaptopodin antibody (1:200) and rabbit anti-podocin antibody (1:300) overnight at 4 °C, followed by incubation with fluorescein cy3-conjμgated anti-rabbit IgG (1:200). Sections were examined by immunofluorescence microscopy. For immunohistochemistry, the 1.5-μm sections were stained with mouse anti-desmin antibody (1:60) at 4 °C overnight, followed by treatment with secondary antibodies. Sections were examined under microscope (Leica DMLB, Wetzlar, Germany).

### Mouse podocyte culture

The conditionally-immortalized mouse podocyte cell line was kindly donated by Professor Karlhans Endlich (University of Greifswald, Griefswald, Mecklenburg-Vorpommern, Germany). They were cultured in RPMI 1640 medium with 10% FCS, 100 U/ml penicillin, and 100 mg/ml streptomycin (Gibco-BRL, Gaithersburg, MD, USA) in a humidified atmosphere of 5% CO_2_. For proliferation, podocytes were cultured in collagen type I-coated flasks in the presence of 10 U/ml mouse recombinant IFN-γ at 33 °C. For differentiation, they were switched to non-IFN-γ medium for 10–14 days at 37 °C.

### Treatment of cultured mouse podocytes

Podocytes *in vitro* were separated into four groups: normal control, PAN treatment, FK506 pretreatment, and CsA pretreatment. They were starved overnight before experiments. The PAN-treatment group was incubated with PAN (50 μg/ml) for 24 h. The pretreatment groups were incubated for 1 h with CsA (5 μg/ml) or FK506 (10 μg/ml) before PAN exposure.

### MTT assay for podocyte proliferation

Podocytes were seeded in 96-well plates at 2 × 10^3^ cells/well and cultured at 37 °C for 10–14 days until fully differentiated. They were, pretreated with CsA or FK506 then treated with PAN for 24 h. Medium was discarded and fresh medium added to each well followed by 20 μl MTT (5 mg/ml). After incubation for 4 h, 150 μl DMSO was added to each well for 30 min. The plates were read at 570 nm on an ELX800NB reader (Biotek, Winooski, VT, USA). Each group had 6 replicate wells.

### Annexin V/propidium iodide (PI) assays for podocyte apoptosis

Each group of podocytes was collected and washed twice with cold PBS, re-suspended in binding buffer at 1 × 10^6^ cells/ml, then AnnexinV-FITC (5 μl) and PI (5 μl) were added to 100 μl of podocyte-containing binding buffer and incubated at room temperature in the dark for 15 min. Apoptosis was analyzed using BD FACS Diva software (BD Biosciences, Franklin Lakes, NJ). Annexin V^+^/PI^−^ podocytes were considered to be early apoptotic and Annexin V^+^/PI^+^ to be late apoptotic cells.

### Transwell migration assays

Transwell cell culture inserts (Cat. 3421; pore size 5 μm; Costar Corp.) were coated with collagen type I, washed once with PBS, and placed in RPMI 1640 medium in the lower compartment; podocytes (1 × 10^4^ cells) were then placed in the inserts. After treatment and migration for 24 h, non-migrated podocytes were removed from the upper membrane, and migrated cells were fixed in cold methanol and stained with crystal violet. Migrated cells were counted under a 10× objective on an inverted microscope (Leica DM4000, Wetzlar, Germany).

### Detection of ATP levels

Intracellular ATP levels were performed with a luciferase-based bioluminescence assay kit according to the manufacturer’s instructions. Podocytes of each group was gathered and centrifugated to remove cell debris, the supernatant was collected. ATP levels was recorded in an Illuminometer (infinite M1000 PRO, TECAN, Switzerland) and expressed as nmol/mg protein.

### Mitochondrial morphology staining

Briefly, podocytes of each group was washed twice with PBS, and incubated with Mito Tracker Red CMXRos (200 nM) for 30 min at 37 °C. After incubation, cells were fixed with cold methanol for 15 min and were visualized using a Nikon A1Ti confocal microscope.

### Mitochondrial Membrane Potential (MMP, ΔΨm) assay for podocytes

Mitochondrial Membrane Potential of podocyte was determined with dual emission mitochondrial dye JC-1(5,5′,6,6′-Tetrachloro-1,1′,3,3′-tetraethyl-imidacarbocyanine iodide) according to the manufacturer’s instructions. JC-1 was able to form aggregates in mitochondria with red fluorescence (emission, 590 nm) at high ΔΨm. In apoptotic cells with low ΔΨm, JC-1 could only form monomer with green fluorescence (emission, 529 nm). Briefly, for flow cytometric detection, podocyte suspension was incubated with JC-1 for 15 minutes at 37 °C in dark. Podocytes were washed twice with JC-1 assay buffer and were quantified by flow cytometry (BD FACS Calibur, Franklin Lakes, NJ, USA) for the determination of cells with green fluorescence. For microscope detection of MMP, podocytes were washed twice with PBS and incubated with JC-1 for 20 minutes at 37 °C in dark and were visualized using a fluorescence microscope.

### Small interfering RNA (siRNA) transfection

The CaN siRNA and control siRNA (CTR siRNA) were synthesized by GenePharma: **CaN siRNA** sequence (**1143:** sense: 5′-GGGUACUUCAGUAUCGAAUTT-3′; antisense: 5′-AUUCGAUACUGAAGUACCCTT-3′. **1539:** sense: 5′-GGCUGUUCGUACUUCUACATT-3′; antisense: 5′-UGUAGAAGUACGAACAGCCTT-3′. **1735**: sense: 5′-CAGUGUUGAAGUACGAGAATT-3′; antisense: 5′-UUCUCGUACUUCAACACUGTT-3′). **Control siRNA** sequence (sense: 5′-UUCUCCGAACGUGUCACGUTT-3′; antisense: GUUCGGAGAATT-3′). Fully differentiated podocytes which were cultured at 37° C for about 10 days were transfected with the siRNA using lipofectamine RNAiMAX transfection protocols. Cells were harvested 48 h after siRNA treatment for Western blot analysis. Functional detection for siRNA-mediated gene silencing was performed at 48 h after siRNA treatment.

### Western blot analysis

For the western blot of cytochrome c, we first separated differentiated podocytes into cytosolic fraction and mitochondrial fraction with Mitochondrial Extraction Kit(Cat:89874; Thermo; USA). Each group of differentiated podocytes was lysed in RIPA buffer with a proteinase inhibitor cocktail from Beyotime Institute of Biotechnology (Shanghai, China). The lysates were boiled with 5 × loading buffer for 5 min at 100 °C. Thirty micrograms of lysate was electrophoresed on 10% SDS-polyacrylamide gels and transferred to PVDF membranes (Millipore, Temecula, CA). Membranes were then blocked with 5% skimmed milk in TBST for 2 h and incubated overnight at 4 °C with different primary antibodies. After washing three times with TBST, the membranes were incubated with 1:3000 HRP-conjμgated secondary antibodies for 2 h. Finally, membranes were analyzed with an enhanced chemiluminescence detection system ChemiDoc MP (Bio-RAD, California, USA). β-actin was used as normalization controls.

### Immunofluorescence staining of cultured podocytes

After culturing on sterile glass coverslips, each group of differentiated podocytes was fixed in 4% paraformaldehyde, permeabilized in 0.3% Triton X-100 for 10 min at room temperature, and then blocked with 5% BSA for 30 min. To stain the cytoskeleton, podocytes were incubated with anti-FITC-phalloidin (1:30) for 60 min at 37 °C. To identify cytoskeletal proteins, podocytes were stained with rabbit anti-synaptopodin (1:50) and rabbit anti-podocin (1:100) overnight at 4 °C. After washing with PBS, cells were labeled with cy3-conjugated anti-rabbit IgG (1:200) and Alexa Fluor 488-conjμgated anti-goat IgG (1:200). DAPI was used to stain nuclei. Podocytes were observed and imaged by fluorescence microscopy (Leica DM4000, Wetzlar, Germany).

### Statistical analysis

Statistical analyses were carried out using SPSS software (version 19.0). All data are presented as mean ± SD. Data from multiple groups were made using one-way ANOVA. Comparisons between groups were made using Student’s *t*-test. P < 0.05 was considered to indicate statistical significance.

## Additional Information

**How to cite this article**: Shen, X. *et al.* Calcineurin inhibitors cyclosporin A and tacrolimus protect against podocyte injury induced by puromycin aminonucleoside in rodent models. *Sci. Rep.* **6**, 32087; doi: 10.1038/srep32087 (2016).

## Supplementary Material

Supplementary Information

## Figures and Tables

**Table 1:** Effects of CsA and FK506 on foot-process width in SD rats.

Foot process width (nm)	10 days	15 days	21 days
Normal control	306 ± 225		
PAN model	1273 ± 1014	960 ± 630	744 ± 401
CsA treatment	650 ± 277ΔΔ	530 ± 234ΔΔ	477 ± 167ΔΔ
FK506 treatment	862 ± 394ΔΔ	395 ± 93ΔΔ	374 ± 90ΔΔ

^ΔΔ^P < 0.01 *vs* PAN model. Quantified foot process width of each group was taken from 3 glomeruli and 3 pictures at 8900 × from each glomerulus.
