# Supplementary material for: Calcineurin inhibitors cyclosporin A and tacrolimus protect against podocyte injury induced by puromycin aminonucleoside in rodent models
Source: Sci Rep. 2016 Sep 1;6:32087. doi: 10.1038/srep32087 (PMC5007516; doi:10.1038/srep32087)
Supplement: Supplementary Information [file srep32087-s1.pdf]

# Supplement Information

## Calcineurin inhibitors cyclosporin A and tacrolimus protect against podocyte injury induced by puromycin aminonucleoside in rodent models

Xiujin Shen[1#], Hong Jiang[1#,*], Meike Ying[1], Zhoutao Xie[1], Xiayu Li[1], Haibing Wang[2], Jie Zhao[1], Chuan Lin[1], Yucheng Wang[1], Shi Feng[1], Jia Shen[1], Chunhua Weng[1], Weiqiang Lin[1], Huiping Wang[1], Qin Zhou[1], Yan Bi[1], Meng Li[1], Lingyan Wang[3], Tongyu Zhu[4], Xiaoru Huang[5], Hui-Yao Lan[5], Jing Zhou[6],Jianghua Chen[1*]

[1]Kidney Disease Center, First Affiliated Hospital, Zhejiang University, School of Medicine; Key Laboratory of Nephropathy, Zhejiang Province, Hangzhou, Zhejiang, China, [2]National Clinical Research Base of Traditional Chinese Medicine, Zhejiang Hospital of Traditional Chinese Medicine, Zhejiang Chinese Medical University, Hangzhou, China, [3]Biomedical Research Center, Zhongshan Hospital, Fudan University, Shanghai, China, [4]Department of Urology, Zhongshan Hospital, Fudan University, Shanghai, China, [5]Li Ka Shing Institute of Health Sciences, and Department of Medicine and Therapeutics, The Chinese University of Hong Kong, Hong Kong, China, [6]Harvard Center for Polycystic Kidney Disease Research and Renal Division, Department of Medicine, Brigham and Women's Hospital, Harvard Medical School, Boston, USA.

[#]These authors contributed equally to this work.

[*]Corresponding author email: annie.jh@163.com, chenjianghua@zju.edu.cn

**Figure legends**

**Fig S1. Effects of CsA and FK506 on podocyte viability in cultured mouse podocytes *in vitro*.** (**A**) MTT assays of podocytes treated with CsA or FK506 alone for 24 h. (**B)** Immunofluorescence of synaptopodin and podocin in cultured mouse podocytes after CsA and FK506 treatment.

**Fig S2. PAN disrupts the podocyte cytoskeleton in cultured mouse podocytes *in vitro*.** (**A**) Immunofluorescence of F-actin showed that PAN disrupted the podocyte cytoskeleton. The green fluorochrome was FITC-phalloidin. Original magnification, ×200. (**B**) Western blot analysis of synaptopodin and podocin levels in PAN-treated podocytes.

**Fig S3. PAN increases apoptosis-related proteins in cultured mouse podocytes *in vitro*.** Western blot analysis of Bax, Bcl-XL, BCL-2 (**A**), and caspase-family protein (**B**) expression after stimulation with 25 μg/ml and 50 μg/ml PAN for 24 h.

**Fig S4. Effects of PAN, CsA, and FK506 on p38, ERK, and JNK signaling pathways in cultured mouse podocytes *in vitro*.** Western blot analysis of p38, ERK, and JNK signaling pathways in PAN, CsA, and FK506-treated podocytes.

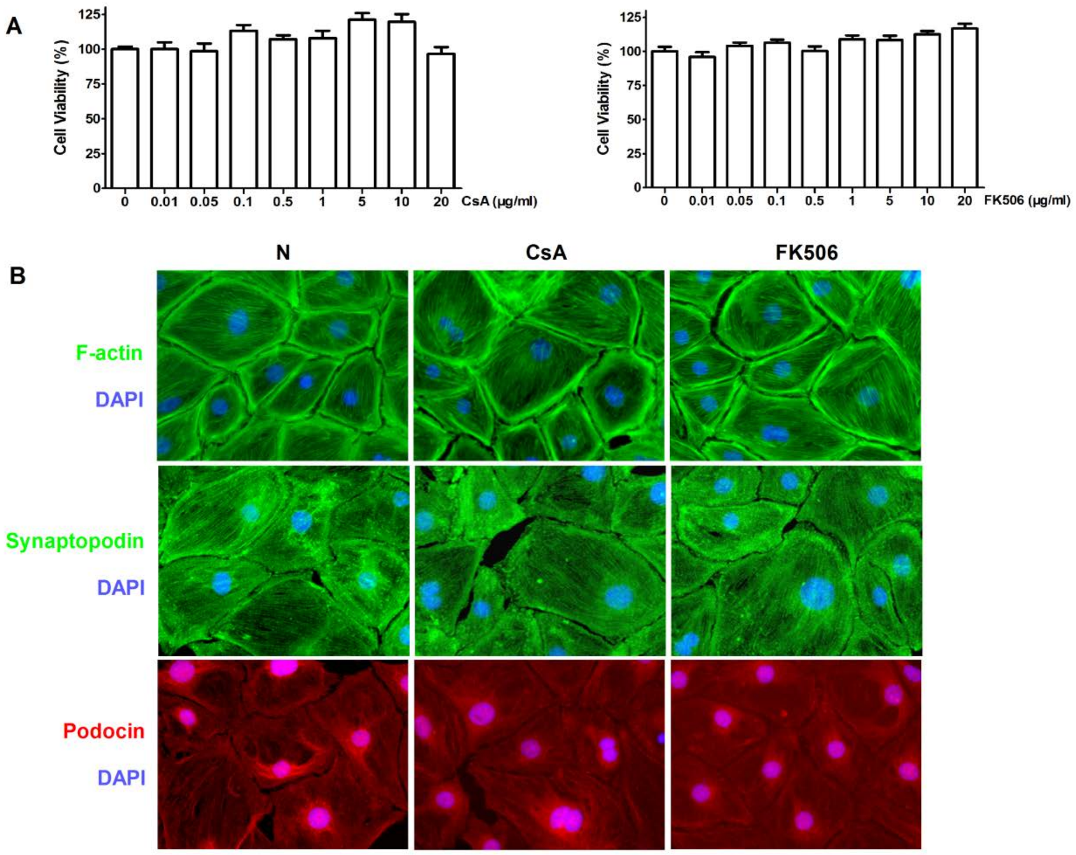


**Fig S1. Effects of CsA and FK506 on podocyte viability in cultured mouse podocytes *in vitro*.** (**A**) MTT assays of podocytes treated with CsA or FK506 alone for 24 h. (**B)** Immunofluorescence of synaptopodin and podocin in cultured mouse podocytes after CsA and FK506 treatment.

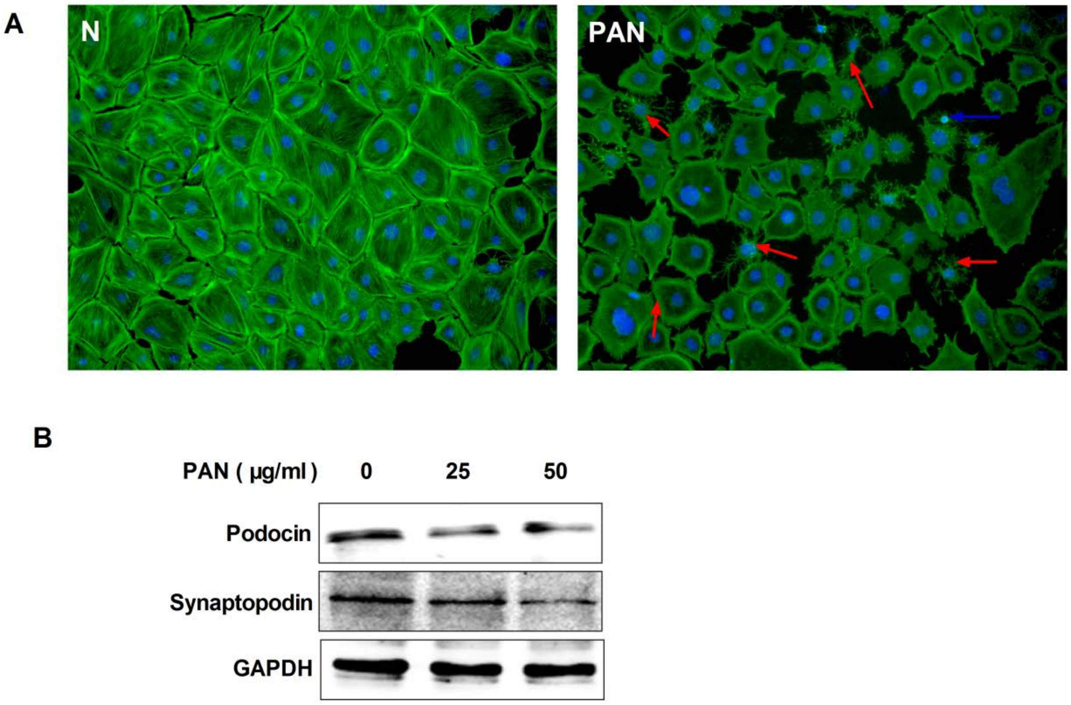


**Fig S2. PAN disrupts the podocyte cytoskeleton in cultured mouse podocytes *in vitro*.** (**A**) Immunofluorescence of F-actin showed that PAN disrupted the podocyte cytoskeleton. The green fluorochrome was FITC-phalloidin. Original magnification, ×200. (**B**) Western blot analysis of synaptopodin and podocin levels in PAN-treated podocytes.

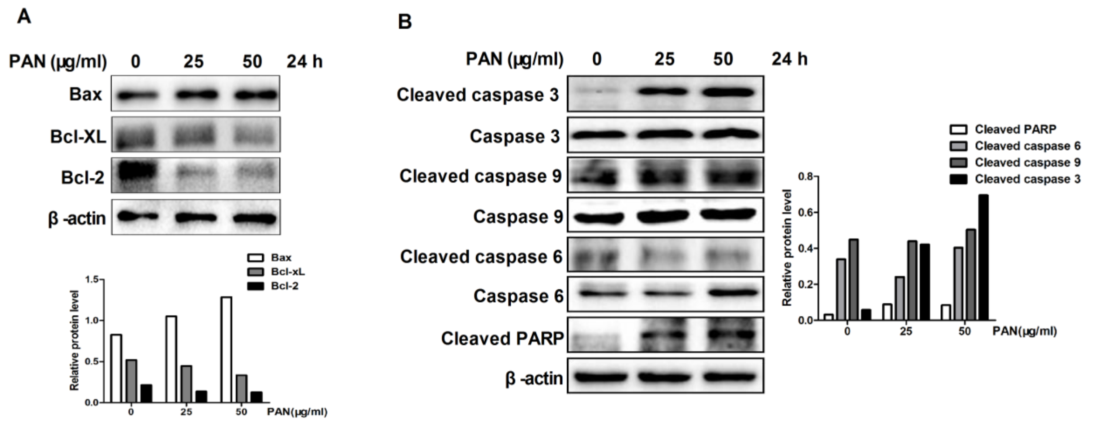


**Fig S3. PAN increases apoptosis-related proteins in cultured mouse podocytes *in vitro*.** Western blot analysis of Bax, Bcl-XL, BCL-2 (**A**), and caspase-family protein (**B**) expression after stimulation with 25 μg/ml and 50 μg/ml PAN for 24 h.

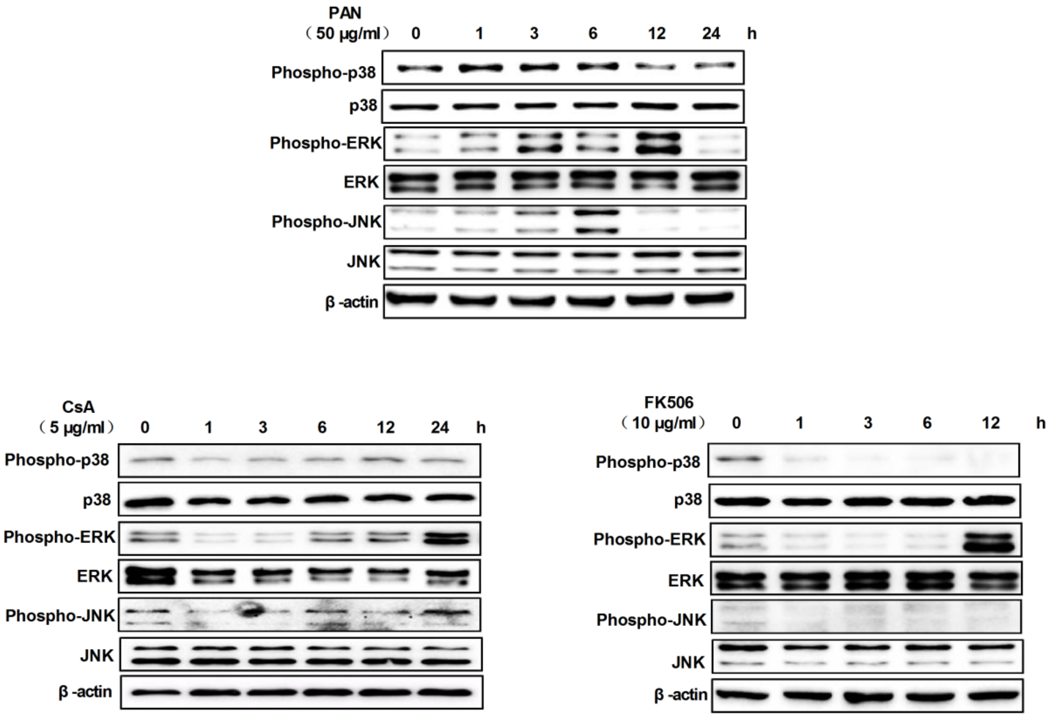


**Fig S4. Effects of PAN, CsA, and FK506 on p38, ERK, and JNK signaling pathways in cultured mouse podocytes *in vitro*.** Western blot analysis of p38, ERK, and JNK signaling pathways in PAN, CsA, and FK506-treated podocytes.

**Table S1**

| Primary antibodies | Host | Dilution and supplier | Product ID | Application |
|---|---|---|---|---|
| ERK | Rabbit | 1:1000; Cell Signaling, Danvers, MA | 4695 | WB |
| phospho-ERK | Rabbit | 1:1000; Cell Signaling, Danvers, MA | 4370 | WB |
| JNK | Rabbit | 1:1000; Cell Signaling, Danvers, MA | 9258 | WB |
| phospho-JNK | Rabbit | 1:1000; Cell Signaling, Danvers, MA | 4668 | WB |
| p38 | Rabbit | 1:1000; Cell Signaling, Danvers, MA | 8690 | WB |
| phospho-p38 | Rabbit | 1:1000; Cell Signaling, Danvers, MA | 4511 | WB |
| Bcl-2 | Rabbit | 1:1000; Cell Signaling, Danvers, MA | 2870 | WB |
| Bcl-xl | Rabbit | 1:1000; Cell Signaling, Danvers, MA | 2764 | WB |
| Bax | Rabbit | 1:1000; Cell Signaling, Danvers, MA | 2772 | WB |
| caspase-3 | Rabbit | 1:1000; Cell Signaling, Danvers, MA | 9662 | WB |
| cleaved caspase-3 | Rabbit | 1:1000; Cell Signaling, Danvers, MA | 9664 | WB |
| caspase-6 | Rabbit | 1:1000; Cell Signaling, Danvers, MA | 9762 | WB |
| cleaved caspase-6 | Rabbit | 1:1000; Cell Signaling, Danvers, MA | 9761 | WB |
| caspase-9 | Rabbit | 1:1000; Cell Signaling, Danvers, MA | 9504 | WB |
| cleaved caspase-9 | Rabbit | 1:1000; Cell Signaling, Danvers, MA | 9509 | WB |
| cleaved PARP | Rabbit | 1:1000; Cell Signaling, Danvers, MA | 9544 | WB |
| desmin | Mouse | 1:60; Zhongshan Golden Bridge Biotechnology, Beijing, China | ZM0091 | IHC |
| WT-1 | Mouse | 1:80; Zhongshan Golden Bridge Biotechnology, Beijing, China | ZM0269 | IHC |
| Synaptopodin | Goat/Rabbit | 1:100/1:1000; Santa Cruz, Dallas, TX | SC-21537/SC50459 | IF/WB |
| podocin | Rabbit | 1:100/1:1000; Sigma, St Louis, MO, USA | P0372 | IF/WB |
| CaN | Rabbit | 1:1000; Santa Cruz, Dallas, TX | SC-9070 | WB |
| β-actin | Mouse | 1:5000; Abcam, Cambridge, MA | Ab6276 | WB |
